# Lung ultrasound in the diagnosis of pneumonia in children: proposal for a new diagnostic algorithm

**DOI:** 10.7717/peerj.1374

**Published:** 2015-11-10

**Authors:** Giulio Iorio, Maria Capasso, Giuseppe De Luca, Salvatore Prisco, Carlo Mancusi, Bruno Laganà, Vincenzo Comune

**Affiliations:** 1 Department of Pediatrics, San Giovanni di Dio Hospital, Azienda Sanitaria Locale Napoli 2 Nord, Frattamaggiore (NA), Italy; 2 District 18, Azienda Sanitaria Locale Caserta, Sant’Arpino (CE), Italy

**Keywords:** Chest radiography, Pneumonia, Community-acquired pneumonia, Lung ultrasound, Lung ultrasonography

## Abstract

**Background.** Despite guideline recommendations, chest radiography (CR) for the diagnosis of community-acquired pneumonia (CAP) in children is commonly used also in mild and/or uncomplicated cases. The aim of this study is to assess the reliability of lung ultrasonography (LUS) as an alternative test in these cases and suggest a new diagnostic algorithm.

**Methods.** We reviewed the medical records of all patients admitted to the pediatric ward from February 1, 2013 to December 31, 2014 with respiratory signs and symptoms. We selected only cases with mild/uncomplicated clinical course and in which CR and LUS were performed within 24 h of each other. The LUS was not part of the required exams recorded in medical records but performed independently. The discharge diagnosis, made only on the basis of history and physical examination, laboratory and instrumental tests, including CR (without LUS), was used as a reference test to compare CR and LUS findings.

**Results.** Of 52 selected medical records CAP diagnosis was confirmed in 29 (55.7%). CR was positive in 25 cases, whereas LUS detected pneumonia in 28 cases. Four patients with negative CR were positive in ultrasound findings. Instead, one patient with negative LUS was positive in radiographic findings. The LUS sensitivity was 96.5% (95% CI [82.2%–99.9%]), specificity of 95.6% (95% CI [78.0%–99.9%]), positive likelihood ratio of 22.2 (95% CI [3.2–151.2]), and negative likelihood ratio of 0.04 (95% CI [0.01–0.25]) for diagnosing pneumonia.

**Conclusion.** LUS can be considered as a valid alternative diagnostic tool of CAP in children and its use must be promoted as a first approach in accordance with our new diagnostic algorithm.

## Introduction

In developed countries the annual incidence of community-acquired pneumonia (CAP) is estimated to be between 34 and 40 per 1000 child years in children younger than five years and represents one of the major causes of morbidity in this age group (Madhi et al., 2013).

The current guidelines suggest that the diagnosis of pneumonia can only be made on the clinical history, respiratory rate, fever, respiratory signs and symptoms reserving the use of radiography only in severe or complicated cases (Harris et al., 2011; Bradley et al., 2011). Despite these latest indications chest radiography (CR) is commonly considered the best choice for the diagnosis of pneumonia among physicians and its execution is also requested for mild cases because of the poor reliability of the history and physical examination (Shah et al., 2010; Ayalon et al., 2013). Furthermore, the question of whether to carry out CR or not in cases of mild or uncomplicated pneumonia depends also, and above all, on the fact that radiological investigation is not entirely harmless (Little, 2003).

In 1986 Weinberg et al. (1986) described a new method of evaluating CAP by the use of lung ultrasonography (LUS). Numerous subsequent studies have shown that it is an accurate, reliable and radiation-free tool in the diagnosis of pneumonia (Parlamento, Copetti & Di Bartolomeo, 2009; Reissig et al., 2012; Iuri, De Candia & Bazzocchi, 2009; Copetti & Cattarossi, 2008; Caiulo et al., 2013). The aim of this study was to evaluate the accuracy of ultrasonography in identifying pneumonia in children and to promote a new diagnostic imaging algorithm for pneumonia with the objective of reducing the “abuse” of CR.

## Materials and Methods

This retrospective study was conducted in the Department of Pediatrics of “San Giovanni di Dio” Hospital, Frattamaggiore (NA), Italy. A total of 1,458 pediatric medical records were reviewed between February 1, 2013 and December 31, 2014. Of these, all cases which were admitted to the pediatric ward with respiratory signs and symptoms were selected. We have excluded all patients with congenital anomalies, immunosuppression, external CR, other comorbidities. We have only included cases in which both CR and LUS were performed within 24 h of each other and with mild/uncomplicated clinical course.

**Figure 1:**
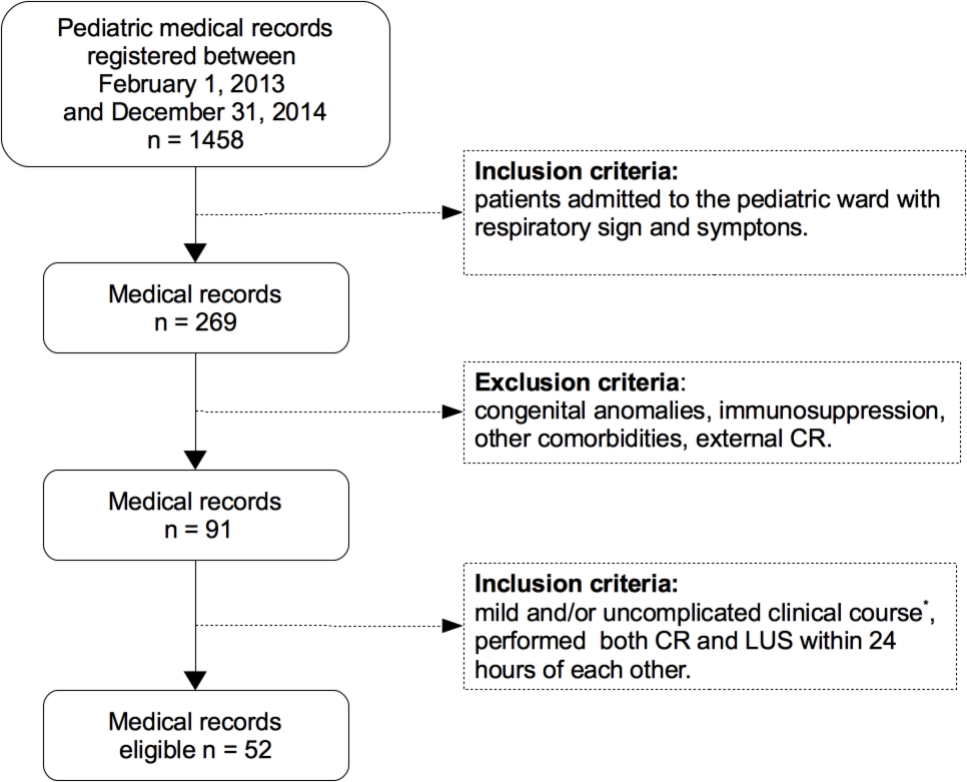
Flowchart medical records selection. ^∗^ Mild disease: Nil or mild increase in effort to breathe, temperature <38.5 °C, respiratory rate <50 breaths/min, mild recession or breathlessness, taking full feeds, no vomiting, oxygen saturation ≥95% in room air according to the criteria of the British Thoracic Society guidelines (Harris et al., 2011). CR, chest radiography; LUS, lung ultrasound.

Finally only 52 medical records met all inclusion and exclusion provided criteria (Fig. 1).

The patients underwent a posterior–anterior CR only, in accordance with the British Thoracic Society (BTS) guidelines (Harris et al., 2011) and the diagnosis of pneumonia was in accordance with the World Health Organization (WHO) criteria for the standardized interpretation of pediatric chest radiographs (World Health Organization Pneumonia Vaccine Trial Investigators’ Group, 2001). The radiologists were blind to the ultrasound findings. The LUS was performed independently either before or after the CR, the two examinations being performed within 24 h of each other. It was not one of the examinations requested by pediatricians and the findings and the images were recorded in order to evaluate the reliability and accuracy of the method. The LUS was always carried out by the same expert operator with a 5–10 MHz linear probe (L38e—Sonosite MicroMaax Systems). The probe was placed perpendicular, oblique and parallel to the ribs in the anterior, lateral and posterior thorax as described by Copetti & Cattarossi (2008) with the patient in the supine position and sitting position to scan the posterior thorax. Color Doppler ultrasound was used to evaluate the vascularity of lung lesion. The sonographer was unaware of the radiograph findings. The CAP was diagnosed in the presence of lung consolidation (hypoechogenic area of varying size and shape with poorly defined borders), air or fluid bronchograms, superficial fluid alveologram, the presence of pleural effusion (Reissig & Kroegel, 2007; Volpicelli et al., 2012).

The final diagnosis of pneumonia was made by pediatricians based on clinical presentations, signs and symptoms such as cough, dyspnea, tachypnea, rales or crackles on auscultation and/or decreased breathing sounds, fever with or without chills, chest and/or abdominal pain, abnormal oxygen saturation, laboratory and instrumental tests, CR findings (without LUS findings) and course compatible with pneumonia defined according to BTS guidelines (Harris et al., 2011).

Lastly, the radiological images which contradicted the ultrasound results were re-evaluated by a senior radiologist during the preparation of this study.

All statistical analysis were performed using PSPP software (Free Software Foundation, Inc.) and MedCalc online software (http://www.medcalc.org/). The study protocol was approved by the Ethics Committee of San Giovanni di Dio Hospital (“Campania Centro”) with approval number 99/2015. All patient records and clinical information were anonymized and de-identified prior to analysis.

## Results

Of a total of 52 medical records the final diagnosis of pneumonia was made in 29 (55.7%). Ages ranged from 2 months to 12.5 years (mean 3.5 y, standard deviation ± 3.1, median 2.6, interquartile 1.0–4.3). The average hospital stay was 5.9 ± 2.5 days and no patients were in intensive care due to disease complications.

Of the 29 patients with pneumonia, chest radiography detected 25 (86.2%) and LUS spotted 28 (96.5%). Instead, of the 23 cases without pneumonia both CR and LUS confirmed negative findings in 22 (specificity of 95.6%). The CR did not identify pneumonia in four patients. One patient with negative findings following LUS was positive with CR. The presence of air bronchograms (multiple hyperechoic inclusions within the pneumonic lesions) was found in 26 of the 28 pneumonias. Instead, the fluid bronchograms, characterized by anechoic or hypoechoic tubular structures in the bronchial tree, were found in 8 of the 28 cases of pneumonia.

Table 1 summarizes the comparison of the CR and LUS results in the diagnosis of pneumonia.

**Table 1:** Comparison of chest radiography and lung ultrasonography results. The diagnosis of pneumonia was made on the basis of history and clinical examination, laboratory and instrumental tests, including chest radiography (without lung ultrasound findings).

	Pneumonia +			Pneumonia −		
	CR+	CR−	Total	CR+	CR−	Total
LUS+	24	4	28	0	1	1
LUS−	1	0	1	1	21	22
Total	25	4	29	1	22	23

**Notes.**

CR chest radiography LUS lung ultrasound + positive - negative

Finally, we calculated the sensitivity, specificity, positive and negative likelihood ratios positive and negative predictive values of CR and LUS (Table 2).

**Table 2:** Diagnostic accuracy of lung ultrasonography and chest radiography in detection of community acquired pneumonia (95% confidence interval).

	Se%	Sp%	LR+	LR−	PPV	NPV
	(95% CI)	(95% CI)	(95% CI)	(95% CI)	(95% CI)	(95% CI)
LUS	96.5	95.6	22.2	0.04	96.5	95.6
	(82.2–99.9)	(78.0–99.9)	(3.2–151.2)	(0.01–0.25)	(82.2–99.9)	(78.0–99.9)
CR	86.2	95.6	19.8	0.14	96.1	84.6
	(68.3–96.1)	(78.0–99.9)	(2.9–135.5)	(0.06–0.36)	(80.3–99.9)	(65.1–95.6)

**Notes.**

Se sensitivity Sp specificity LR+ positive likelihood ratio LR− negative likelihood ratio PPV positive predictive value NPV negative predictive value CI confidence interval CR chest radiography LUS lung ultrasound

## Discussion

The diagnosis of pneumonia can also be made without resorting to the chest X-ray (Harris et al., 2011; Bradley et al., 2011). The main limitations of radiography are firstly the risk of damage from ionizing radiation with a greater risk than adults because children have more rapidly dividing cells and increased life expectancy (Ait-Ali et al., 2010; Miller, 1995), the great variability in the interpretation of the examination (Johnson & Kline, 2010; Williams et al., 2013), no substantial impact on clinical outcomes (Swingler, Hussey & Zwarenstein, 1998; Swingler, 2009; Levinsky et al., 2013).

Although for a long time it was thought that lung ultrasound was rendered impossible by the air content, numerous studies in the literature in adults (Parlamento, Copetti & Di Bartolomeo, 2009; Reissig et al., 2012; Lichtenstein et al., 2004; Blaivas, 2012; Xirouchaki et al., 2011) and later in children have demonstrated its efficacy for the diagnosis of pneumonia with a sensitivity and a specificity superior to chest X-ray (Iuri, De Candia & Bazzocchi, 2009; Copetti & Cattarossi, 2008; Caiulo et al., 2013; Reali et al., 2014; Kurian et al., 2009). The validity of the method has also been confirmed in the neonatal period and not only for the diagnosis of pneumonia (Liu et al., 2014; Raimondi et al., 2012; Piastra et al., 2014; Raimondi et al., 2014).

The sonographic signs of pneumonia are the presence of a subpleural hypoechoic region with hyperechoic spots of variable size (air bronchograms), fluid bronchograms, confluent B-lines, superficial fluid alveologram, a vascular tree-shaped pattern (Reissig & Kroegel, 2007; Volpicelli et al., 2012). In our work the presence of the various sonographic signs has an incidence similar to the data in the literature (Reissig et al., 2012; Reissig & Kroegel, 2007). Air bronchograms, due to the presence of trapped air in an airway, were identified in 92.8%. Fluid bronchograms were found in 28.5%, a high percentage due to the fact that in pediatric age postobstructive pneumonia is frequent, while the superficial fluid alveolograms were found in 75.0%.

**Figure 2:**
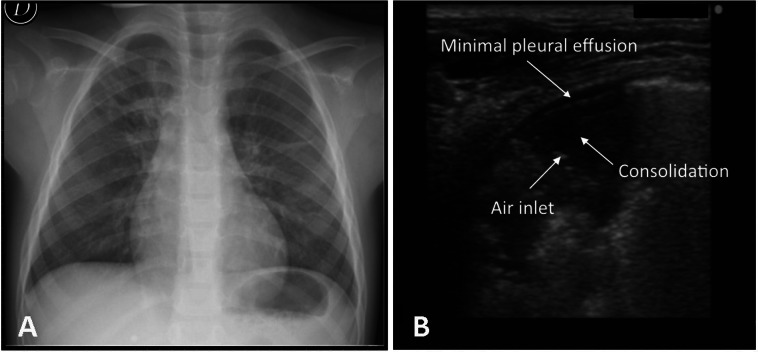
One case of negative chest X-ray and positive lung ultrasound. Negative chest X-ray result for a 4 year old female (A) and evidence of pneumonia in the posterior basal retrocardiac region of left lung by lung ultrasound (B).

In our study the CR did not identify four cases of pneumonia which instead were detected by ultrasonography (Fig. 2). The failure of radiological diagnosis is linked to the position of lesion, like the retrocardiac (2 cases) or juxta-diaphragmatic region (1 case), and radiolucency in the early stages of a pneumonic process (1 case). Further possible reasons could be the high variability of interpretation (Johnson & Kline, 2010; Williams et al., 2013) and the limitations of radiographic resolution less than 1 cm (Raoof et al., 2012) (Fig. 2).

**Figure 3:**
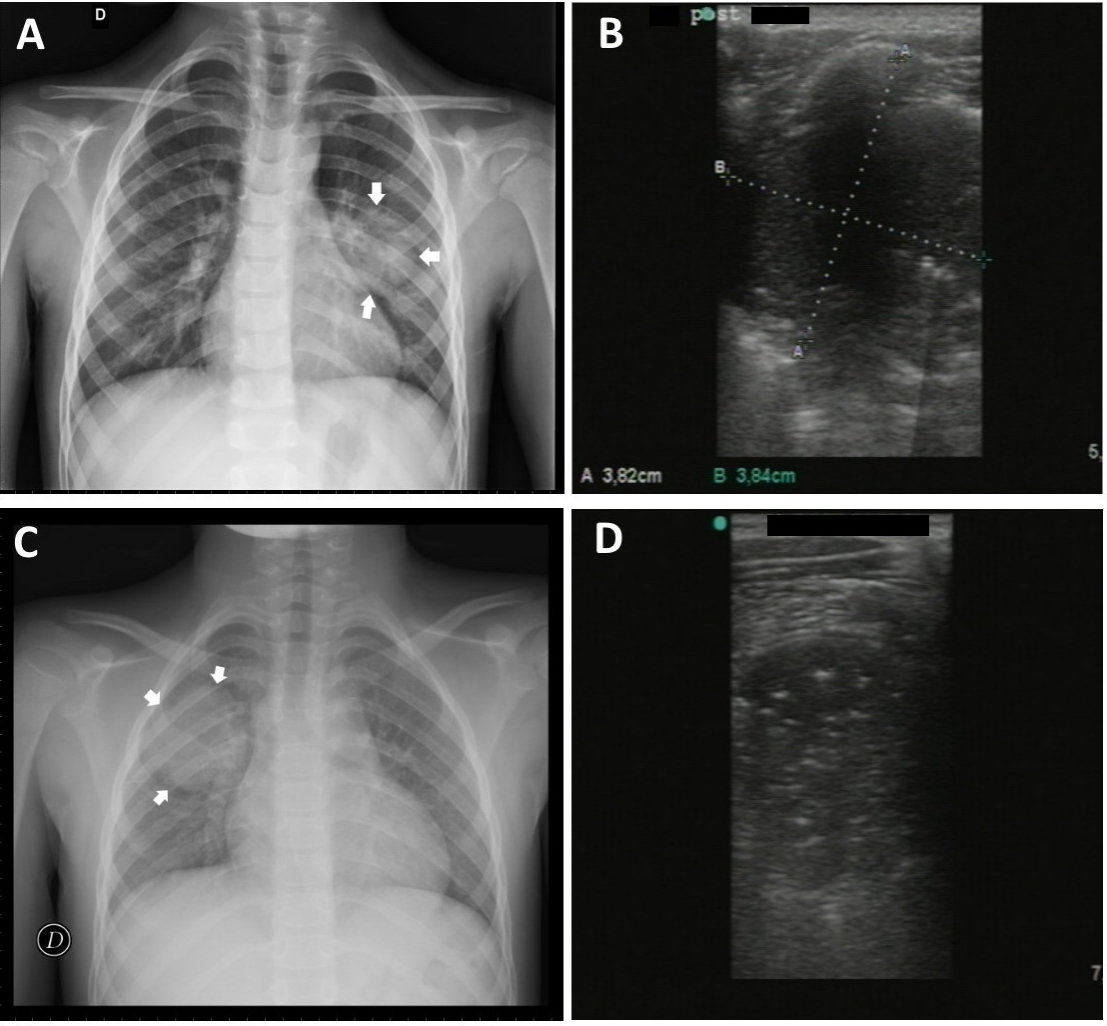
Round pneumonia. Case 1. 5 year old male with evidence of round pneumonia by chest X-ray in middle region of the left lung (A) duly detected by lung ultrasound (B) Case 2. 8 year old male with round pneumonia in middle/upper region of right lung by chest rx (C) and corresponding ultrasound image (D).

One case of pneumonia which was not seen by LUS was instead identified by CR. In this case the CR was negative following a subsequent evaluation by the senior radiologist made during the preparation of this study. However, the real failure in the ultrasound diagnosis of pneumonia can be attributed either to not reaching the lesion to the pleural line or to not being able to easily explore the supraclavicular region and/or the area covered by the scapula. In the latter case it is sufficient to change the position of the upper limb to discover the region covered by the scapula. Lichtenstein & Mezière (2008) confirmed that acute alveolar consolidation in adults reaches the pleura in 98.5% of cases. Round pneumonia must be considered in children as possible injury that does not extend to the pleura. Its prevalence in adults is less than 1% whereas in children it could be greater because of underdeveloped pathways of collateral ventilation (the pores of Kohn, channels of Lambert) (Celebi & Hacimustafaoglu, 2008). Moreover, these lesions are most common in the peripheral areas of lung which come more easily into contact with the pleural line (Kim & Donnelly, 2007). A recent pilot study of Corradi et al. (2015) showed that the quantitative lung ultrasonography could be a new tool in the diagnosis of CAP distant from the pleural line. In our study two cases of round pneumonia were detected regularly by LUS (Fig. 3).

Finally, the ultrasound false-positive case was due to the presence of a small subpleural consolidation (<1 cm) not visible in X-ray. Instead, the radiographic false-positive result was due to confusing thymus for a pneumonic opacity.

In another case not reported in the study because the ultrasound examination was carried out three days after the negative chest X-ray, LUS diagnosed a segmental lung consolidation in the axillary basal right region. The opportunity to also repeat the exam a short time later is an exclusive feature of ultrasound examination. A further advantage of ultrasound is as a follow-up exam for pneumonia and in particular to monitor the progress in improvement following therapy.

Some limitations of this study are clear. First, it is a retrospective study and the number of cases is low. The patients performed the radiological and the ultrasound examination at different times and within 24 h of each other whereas some injuries may improve or worsen rapidly within this timeframe. Second, the radiologist was whoever was on duty at the time while the lung ultrasound was always performed by the one same experienced operator (bias which favors LUS). Both the radiologists and sonographer knew the medical history but were unaware of each other’s respective reports. Third, we have considered the clinical and instrumental examinations including CR as reference for the diagnosis of pneumonia. Computed tomography should be used as a gold standard reference but in the clinical cases examined, the computed tomography was never requested as a diagnostic test. Anyway, our results are similar to data in previous prospective studies (Caiulo et al., 2013; Reali et al., 2014).

**Figure 4:**
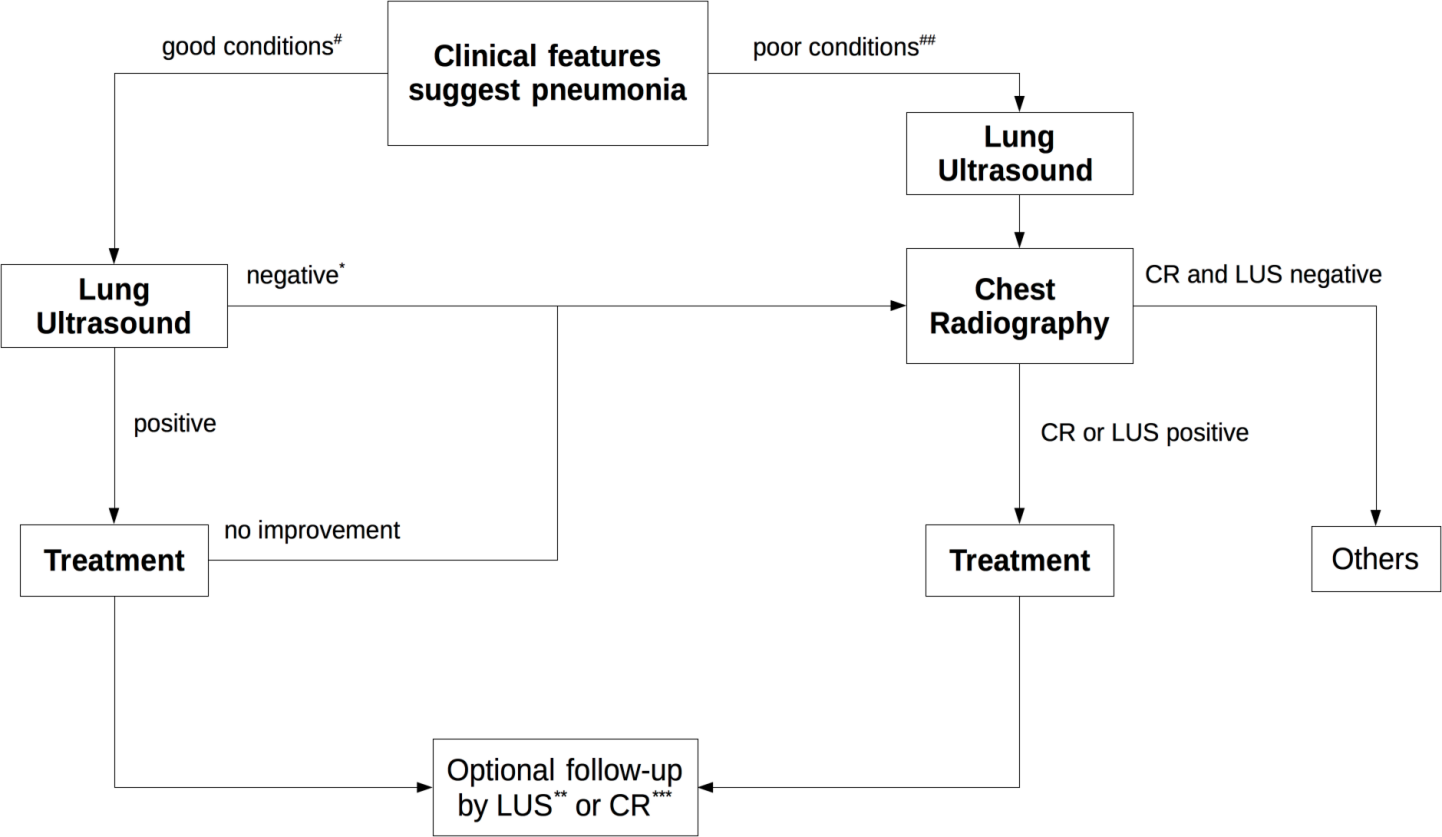
New diagnostic imaging algorithm for diagnosis of pneumonia. #Nil or mild increase in effort to breathe, temperature <38.5 C, respiratory rate <50 breaths/min, mild recession or breathlessness, taking full feeds, no vomiting, oxygen saturation ≥95% in room air. ## Temperature >38.58 C, respiratory rate >70 breaths/min, moderate to severe recession, nasal flaring, cyanosis, intermittent apnoea, grunting respiration, not feeding, tachycardia, capillary refill time >2 s, oxygen saturation ≤95% in room air. ^∗^ If conditions are good after 24–48 h the lung ultrasound can also be repeated or improvement after therapy can be checked. ^∗∗^ In all cases. ^∗∗∗^ In cases provided for by guidelines. CR, Chest Rx; LUS, Lung Ultrasound.

Even with the above limitations, LUS showed a high ability to detect pneumonia in children. On the basis of our study and the scientific literature about the reliability of ultrasound examination (Nazerian et al., 2015; Shah, Tunik & Tsung, 2013), the most recent meta-analysis (Chavez et al., 2014; Pereda et al., 2015), in accordance with limiting the use of X-rays only in patients with serious conditions, we suggest the use of lung ultrasound in the first instance by following the new algorithm shown in Fig. 4.

In cases where clinical pneumonia is suspected, if the conditions are good, a lung ultrasound is performed as the first step. If the ultrasound examination does not confirm pneumonia, after 24–48 h LUS can be repeated or improvement after therapy can be checked otherwise CR can be performed. In poor conditions LUS and CR can be requested and, if both findings are negative, another diagnosis can be considered. Finally, after diagnosis of pneumonia, LUS can be carried out optionally in all cases or CR only in cases provided for by the guidelines. It can be noted from the algorithm that if LUS is excluded, current guidelines are followed. We suggest simply to include lung ultrasonography as a first step without changing the guideline recommendations.

## Conclusions

In conclusion, lung ultrasound shows high reliability and accuracy in the detection of pneumonia, the possibility of a follow-up until the complete resolution of lung injury, without exposure to ionizing radiation. It does not require sedation and can be repeated at any time. If necessary, CR can always be performed but we believe in the routine use (first approach) of LUS for children where pneumonia is suspected in accordance with our diagnostic algorithm with the aim of limiting the use of CR only to the serious cases provided for by the guidelines and reserving the use of a diagnostic imaging without risks such as lung ultrasound in all other cases.

## Supplemental Information

10.7717/peerj.1374/supp-1 Supplemental Information 1 Raw data
